# Bis{2,4-dibromo-6-[(2-phenyl­eth­yl)imino­meth­yl]phenolato-κ^2^ *N*,*O*}cobalt(II)

**DOI:** 10.1107/S160053681104459X

**Published:** 2011-10-29

**Authors:** Yanli Yin, Jinrong Wang, Yongliang Zhao, Liang Huang

**Affiliations:** a College of Biological Engineering, Henan University of Technology, Zhengzhou, Henan 450001, People’s Republic of China

## Abstract

In the title complex, [Co(C_15_H_12_Br_2_NO)_2_], the Co^II^ atom is four-coordinated by two *N*,*O*-bidentate chelate Schiff base ligands, displaying a flattened tetra­hedral coordination environment. The Co^II^ atom occupies a special position on a twofold rotation axis. In the crystal, mol­ecules are linked *via* weak C—H⋯Br inter­actions.

## Related literature

For background to vitamin B12, see: Randaccio *et al.* (2010 ▶). For the anti­tumour activity of Schiff base–metal complexes, see: Ren *et al.* (2002 ▶) and for their anti-microbial activity, see: Panneerselvam *et al.* (2005 ▶). For related structures, see: Chen *et al.* (2010 ▶); Li *et al.* (2010 ▶); Jiang *et al.* (2008 ▶); For standard bond lengths, see: Allen *et al.* (1987 ▶).
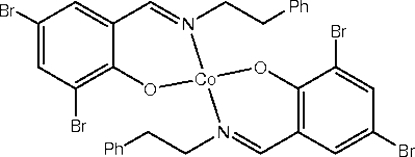


## Experimental

### 

#### Crystal data

[Co(C_15_H_12_Br_2_NO)_2_]
*M* *_r_* = 823.08
Monoclinic, 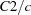

*a* = 22.5087 (16) Å
*b* = 4.8717 (4) Å
*c* = 28.864 (2) Å
β = 111.505 (1)°
*V* = 2944.8 (4) Å^3^
*Z* = 4
Mo *K*α radiation
μ = 6.04 mm^−1^
*T* = 291 K
0.24 × 0.23 × 0.22 mm

#### Data collection

Bruker SMART APEX CCD diffractometer
Absorption correction: multi-scan (*SADABS*; Bruker, 2000 ▶) *T*_min_ = 0.325, *T*_max_ = 0.350
14453 measured reflections
2876 independent reflections
2478 reflections with *I* > 2σ(*I*)
*R*_int_ = 0.031

#### Refinement

*R*[*F* ^2^ > 2σ(*F* ^2^)] = 0.032
*wR*(*F* ^2^) = 0.108
*S* = 1.01
2876 reflections
177 parameters
H-atom parameters constrained
Δρ_max_ = 0.79 e Å^−3^
Δρ_min_ = −0.78 e Å^−3^

### 

Data collection: *SMART* (Bruker, 2000 ▶); cell refinement: *SAINT-Plus* (Bruker, 2000 ▶); data reduction: *SAINT-Plus*; program(s) used to solve structure: *SHELXS97* (Sheldrick, 2008 ▶); program(s) used to refine structure: *SHELXTL* (Sheldrick, 2008 ▶); molecular graphics: *SHELXTL*; software used to prepare material for publication: *SHELXTL*.

## Supplementary Material

Crystal structure: contains datablock(s) global, I. DOI: 10.1107/S160053681104459X/br2176sup1.cif

Structure factors: contains datablock(s) I. DOI: 10.1107/S160053681104459X/br2176Isup2.hkl

Additional supplementary materials:  crystallographic information; 3D view; checkCIF report

## Figures and Tables

**Table 1:** Selected bond lengths (Å)

Co1—O1	1.916 (2)
Co1—N1	1.986 (3)

**Table 2:** Hydrogen-bond geometry (Å, °)

*D*—H⋯*A*	*D*—H	H⋯*A*	*D*⋯*A*	*D*—H⋯*A*
C7—H7⋯Br2^i^	0.93	3.01	3.940 (3)	173
C8—H8*B*⋯Br1^ii^	0.97	2.93	3.814 (3)	151
C9—H9*B*⋯Br2^iii^	0.97	2.94	3.854 (3)	157

Symmetry codes: (i) 
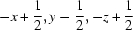
; (ii) 
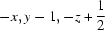
; (iii) 
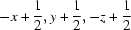
.

**Fig. 1.**
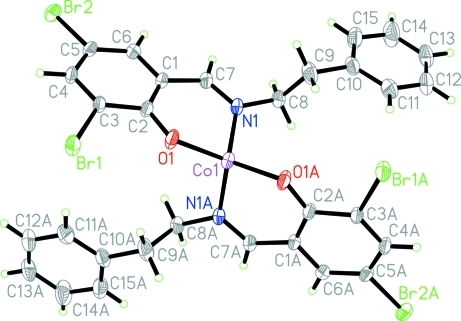
The structure of the title compound, with the atom numbering scheme of the unique atoms (30% probability ellipsoids).

**Fig. 2.**
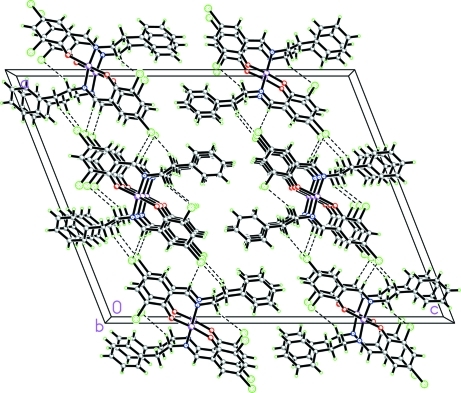
Partial packing view showing the chain formed through weak C–H···Br interactions.

## supplementary crystallographic information

### Comment

Cobalt is an important life-required element. For example, vitamin B12, also called cobalamin, is a water soluble vitamin with a key role in the normal functioning of the brain and nervous system, and for the formation of blood (Randaccio *et al.*, 2010). In addition, the Schiff base metal complexes generally possess antitumour activities (Ren *et al.*, 2002) and antimicrobial activities (Panneerselvam *et al.*, 2005). By taking the biological importance of element cobalt into account, we synthesized the title complex with the bidentate N,O-donor Schiff base ligands (Scheme I).

In the title compound, the Co^II^ atom occupies a special position on a twofold rotation axis to form a distorted tetrahedral coordination sphere. Cobalt(II) atom is four-coordinated by two imino N atoms and two phenolic O atoms from two bidentate Schiff-base ligands derived from the condensation of 3,5-dibromosalicylaldehyde and 2-phenylethylamine (Fig. 1). All bond lengths are within normal ranges (Allen *et al.*, 1987). The C7═N1 bond length of 1.284 (4) Å is within the range of 1.256 (14)–1.310 (15) Å observed in the analogous tetrahedral Co(II) species  (Chen *et al.*, 2010; Li *et al.*, 2010). The Co–O and Co–N bond distances of 1.916 (2) and 1.986 (3) Å are also similar to those of 1.935 (2) and 2.006 (3) Å previously reported in the related cobalt(II) complex of a Schiff base ligand derived from the condensation of 3,5-dibromosalicylaldehyde and benzylamine (Jiang *et al.*, 2008).

In the crystal structure, the molecules are linked *via* weak C–H···Br interactions (Fig.2).

### Experimental

3,5-Dibromosalicylaldehyde (560 mg, 2 mmol) and 2-phenylethylamine (242 mg, 2 mmol) were dissolved in a methanol solution (25 mL).The mixture was stirred at room temperature for 1 h to give an orange solution, which was added to a methanol solution (15 mL) of Co(NO_3_)_2_.6H_2_O (280 mg, 1 mmol). The mixture was stirred for another 25 min at room temperature to give a red solution and then filtered. The filtrate was kept in air for 7 days, forming  red blocky crystals. The crystals were isolated and dried in a vacuum desiccator containing anhydrous CaCl_2_, in about 64% yield. Anal. Calcd for C_30_H_24_Br_4_CoN_2_O_2_: C, 43.78; H, 2.94; N, 3.40. Found: C, 43.66; H, 2.99; N, 3.31%. IR (KBr, cm^-1^): 3423, 2909, 2361, 1614, 1502, 1433, 1410, 1310, 1210, 1152, 865, 749, 703, 486, 437.

### Refinement

All the H atoms were placed in geometrically idealized positions and constrained to ride on their parent atoms, with C—H distances of 0.93 and 0.97 Å, and with *U*_iso_(H) = 1.2*U*_eq_(carrier).

### Crystal data

**Table:**

[Co(C_15_H_12_Br_2_NO)_2_]	*F*(000) = 1604
*M**_r_* = 823.08	*D*_x_ = 1.857 Mg m^−^^3^
Monoclinic, *C*2/*c*	Mo *K*α radiation, λ = 0.71073 Å
Hall symbol: -C 2yc	Cell parameters from 5864 reflections
*a* = 22.5087 (16) Å	θ = 2.9–28.1°
*b* = 4.8717 (4) Å	µ = 6.04 mm^−^^1^
*c* = 28.864 (2) Å	*T* = 291 K
β = 111.505 (1)°	Block, red
*V* = 2944.8 (4) Å^3^	0.24 × 0.23 × 0.22 mm
*Z* = 4	

### Data collection

**Table:**

Bruker SMART APEX CCD diffractometer	2876 independent reflections
Radiation source: fine-focus sealed tube	2478 reflections with *I* > 2σ(*I*)
graphite	*R*_int_ = 0.031
φ and ω scans	θ_max_ = 26.0°, θ_min_ = 1.9°
Absorption correction: multi-scan (*SADABS*; Bruker, 2000)	*h* = −27→27
*T*_min_ = 0.325, *T*_max_ = 0.350	*k* = −6→6
14453 measured reflections	*l* = −35→35

### Refinement

**Table:**

Refinement on *F*^2^	Primary atom site location: structure-invariant direct methods
Least-squares matrix: full	Secondary atom site location: difference Fourier map
*R*[*F*^2^ > 2σ(*F*^2^)] = 0.032	Hydrogen site location: inferred from neighbouring sites
*wR*(*F*^2^) = 0.108	H-atom parameters constrained
*S* = 1.01	*w* = 1/[σ^2^(*F*_o_^2^) + (0.082*P*)^2^ + 0.812*P*] where *P* = (*F*_o_^2^ + 2*F*_c_^2^)/3
2876 reflections	(Δ/σ)_max_ < 0.001
177 parameters	Δρ_max_ = 0.79 e Å^−^^3^
0 restraints	Δρ_min_ = −0.78 e Å^−^^3^

### Special details

**Table:**

Geometry. All esds (except the esd in the dihedral angle between two l.s. planes) are estimated using the full covariance matrix. The cell esds are taken into account individually in the estimation of esds in distances, angles and torsion angles; correlations between esds in cell parameters are only used when they are defined by crystal symmetry. An approximate (isotropic) treatment of cell esds is used for estimating esds involving l.s. planes.
Refinement. Refinement of F^2^ against ALL reflections. The weighted R-factor wR and goodness of fit S are based on F^2^, conventional R-factors R are based on F, with F set to zero for negative F^2^. The threshold expression of F^2^ > 2sigma(F^2^) is used only for calculating R-factors(gt) etc. and is not relevant to the choice of reflections for refinement. R-factors based on F^2^ are statistically about twice as large as those based on F, and R- factors based on ALL data will be even larger.

### Fractional atomic coordinates and isotropic or equivalent isotropic displacement parameters (Å^2^)

**Table:**

	*x*	*y*	*z*	*U*_iso_*/*U*_eq_	
C1	0.12749 (15)	0.3862 (7)	0.22685 (12)	0.0389 (7)	
C2	0.08497 (15)	0.5880 (6)	0.19768 (11)	0.0367 (7)	
C3	0.09703 (15)	0.6772 (6)	0.15481 (11)	0.0365 (6)	
C4	0.14508 (15)	0.5753 (6)	0.14172 (12)	0.0409 (7)	
H4	0.1509	0.6379	0.1132	0.049*	
C5	0.18509 (15)	0.3773 (7)	0.17151 (12)	0.0430 (7)	
C6	0.17739 (16)	0.2844 (7)	0.21350 (12)	0.0435 (8)	
H6	0.2052	0.1534	0.2333	0.052*	
C7	0.12310 (17)	0.2717 (7)	0.27173 (12)	0.0431 (7)	
H7	0.1561	0.1560	0.2903	0.052*	
C8	0.08535 (19)	0.1846 (8)	0.33657 (12)	0.0492 (8)	
H8A	0.1125	0.0239	0.3418	0.059*	
H8B	0.0438	0.1257	0.3358	0.059*	
C9	0.1142 (2)	0.3836 (9)	0.37889 (14)	0.0656 (11)	
H9A	0.0876	0.5464	0.3729	0.079*	
H9B	0.1560	0.4390	0.3799	0.079*	
C10	0.12060 (19)	0.2619 (7)	0.42885 (13)	0.0503 (9)	
C11	0.0790 (2)	0.3341 (10)	0.45192 (14)	0.0613 (10)	
H11	0.0479	0.4661	0.4374	0.074*	
C12	0.0828 (3)	0.2136 (10)	0.49615 (16)	0.0701 (12)	
H12	0.0536	0.2616	0.5107	0.084*	
C13	0.1281 (3)	0.0287 (10)	0.51828 (15)	0.0702 (12)	
H13	0.1309	−0.0479	0.5485	0.084*	
C14	0.1703 (3)	−0.0485 (13)	0.49693 (19)	0.0972 (19)	
H14	0.2012	−0.1803	0.5121	0.117*	
C15	0.1668 (2)	0.0710 (11)	0.45224 (17)	0.0773 (14)	
H15	0.1962	0.0209	0.4380	0.093*	
Br1	0.040636 (17)	0.94006 (7)	0.113456 (12)	0.04757 (15)	
Br2	0.250012 (17)	0.22456 (9)	0.151646 (14)	0.05805 (16)	
Co1	0.0000	0.52246 (15)	0.2500	0.04521 (19)	
N1	0.07834 (13)	0.3134 (6)	0.28853 (10)	0.0425 (6)	
O1	0.03706 (12)	0.6888 (5)	0.20677 (9)	0.0468 (6)	

### Atomic displacement parameters (Å^2^)

**Table:**

	*U*^11^	*U*^22^	*U*^33^	*U*^12^	*U*^13^	*U*^23^
C1	0.0389 (16)	0.0510 (18)	0.0320 (16)	−0.0033 (13)	0.0192 (13)	−0.0013 (13)
C2	0.0394 (16)	0.0439 (17)	0.0338 (16)	−0.0048 (12)	0.0219 (13)	−0.0012 (12)
C3	0.0406 (16)	0.0432 (16)	0.0303 (15)	−0.0029 (13)	0.0182 (13)	0.0005 (12)
C4	0.0460 (18)	0.0523 (19)	0.0324 (16)	−0.0078 (15)	0.0239 (14)	−0.0023 (13)
C5	0.0360 (16)	0.0589 (19)	0.0414 (18)	−0.0039 (14)	0.0226 (14)	−0.0086 (15)
C6	0.0364 (16)	0.059 (2)	0.0370 (17)	0.0031 (14)	0.0155 (14)	0.0028 (14)
C7	0.0444 (18)	0.0548 (19)	0.0336 (16)	0.0020 (14)	0.0184 (14)	0.0063 (14)
C8	0.054 (2)	0.064 (2)	0.0345 (17)	−0.0061 (17)	0.0213 (15)	0.0100 (15)
C9	0.091 (3)	0.069 (2)	0.038 (2)	−0.021 (2)	0.026 (2)	0.0042 (17)
C10	0.062 (2)	0.057 (2)	0.0324 (17)	−0.0141 (17)	0.0174 (16)	−0.0011 (14)
C11	0.067 (2)	0.074 (3)	0.040 (2)	0.005 (2)	0.0150 (18)	0.0093 (18)
C12	0.089 (3)	0.087 (3)	0.044 (2)	0.001 (3)	0.035 (2)	−0.004 (2)
C13	0.095 (3)	0.083 (3)	0.036 (2)	0.004 (3)	0.027 (2)	0.0095 (19)
C14	0.114 (4)	0.133 (5)	0.050 (3)	0.052 (4)	0.036 (3)	0.032 (3)
C15	0.076 (3)	0.116 (4)	0.047 (2)	0.022 (3)	0.032 (2)	0.013 (2)
Br1	0.0584 (2)	0.0515 (2)	0.0413 (2)	0.00711 (15)	0.02841 (17)	0.00863 (13)
Br2	0.0447 (2)	0.0830 (3)	0.0575 (3)	0.00539 (17)	0.03169 (19)	−0.00833 (18)
Co1	0.0441 (4)	0.0638 (4)	0.0359 (4)	0.000	0.0243 (3)	0.000
N1	0.0470 (16)	0.0554 (16)	0.0312 (14)	−0.0017 (13)	0.0216 (12)	0.0052 (12)
O1	0.0522 (14)	0.0575 (14)	0.0442 (13)	0.0103 (11)	0.0337 (11)	0.0089 (11)

### Geometric parameters (Å, °)

**Table:**

C1—C6	1.405 (5)	C9—C10	1.516 (5)
C1—C2	1.415 (5)	C9—H9A	0.9700
C1—C7	1.447 (4)	C9—H9B	0.9700
C2—O1	1.296 (4)	C10—C15	1.374 (6)
C2—C3	1.428 (4)	C10—C11	1.378 (6)
C3—C4	1.363 (4)	C11—C12	1.379 (6)
C3—Br1	1.888 (3)	C11—H11	0.9300
C4—C5	1.383 (5)	C12—C13	1.335 (7)
C4—H4	0.9300	C12—H12	0.9300
C5—C6	1.363 (5)	C13—C14	1.360 (7)
C5—Br2	1.906 (3)	C13—H13	0.9300
C6—H6	0.9300	C14—C15	1.391 (7)
C7—N1	1.284 (4)	C14—H14	0.9300
C7—H7	0.9300	C15—H15	0.9300
C8—N1	1.477 (4)	Co1—O1	1.916 (2)
C8—C9	1.507 (5)	Co1—O1^i^	1.916 (2)
C8—H8A	0.9700	Co1—N1	1.986 (3)
C8—H8B	0.9700	Co1—N1^i^	1.986 (3)
			
C6—C1—C2	121.1 (3)	C10—C9—H9B	109.1
C6—C1—C7	115.7 (3)	H9A—C9—H9B	107.8
C2—C1—C7	123.2 (3)	C15—C10—C11	117.4 (3)
O1—C2—C1	125.0 (3)	C15—C10—C9	121.6 (4)
O1—C2—C3	119.8 (3)	C11—C10—C9	121.0 (4)
C1—C2—C3	115.2 (3)	C10—C11—C12	121.1 (4)
C4—C3—C2	123.5 (3)	C10—C11—H11	119.5
C4—C3—Br1	119.3 (2)	C12—C11—H11	119.5
C2—C3—Br1	117.2 (2)	C13—C12—C11	120.5 (4)
C3—C4—C5	118.9 (3)	C13—C12—H12	119.8
C3—C4—H4	120.5	C11—C12—H12	119.8
C5—C4—H4	120.5	C12—C13—C14	120.5 (4)
C6—C5—C4	121.2 (3)	C12—C13—H13	119.8
C6—C5—Br2	120.0 (3)	C14—C13—H13	119.8
C4—C5—Br2	118.8 (2)	C13—C14—C15	119.5 (5)
C5—C6—C1	120.1 (3)	C13—C14—H14	120.2
C5—C6—H6	119.9	C15—C14—H14	120.2
C1—C6—H6	119.9	C10—C15—C14	121.0 (5)
N1—C7—C1	126.7 (3)	C10—C15—H15	119.5
N1—C7—H7	116.7	C14—C15—H15	119.5
C1—C7—H7	116.7	O1—Co1—O1^i^	129.98 (15)
N1—C8—C9	110.7 (3)	O1—Co1—N1	94.15 (10)
N1—C8—H8A	109.5	O1^i^—Co1—N1	111.18 (11)
C9—C8—H8A	109.5	O1—Co1—N1^i^	111.18 (11)
N1—C8—H8B	109.5	O1^i^—Co1—N1^i^	94.14 (10)
C9—C8—H8B	109.5	N1—Co1—N1^i^	118.31 (17)
H8A—C8—H8B	108.1	C7—N1—C8	117.3 (3)
C8—C9—C10	112.5 (3)	C7—N1—Co1	121.9 (2)
C8—C9—H9A	109.1	C8—N1—Co1	120.7 (2)
C10—C9—H9A	109.1	C2—O1—Co1	124.4 (2)
C8—C9—H9B	109.1		

Symmetry codes: (i) −*x*, *y*, −*z*+1/2.

### Hydrogen-bond geometry (Å, °)

**Table:**

*D*—H···*A*	*D*—H	H···*A*	*D*···*A*	*D*—H···*A*
C7—H7···Br2^ii^	0.93	3.01	3.940 (3)	173.
C8—H8B···Br1^iii^	0.97	2.93	3.814 (3)	151.
C9—H9B···Br2^iv^	0.97	2.94	3.854 (3)	157.

Symmetry codes: (ii) −*x*+1/2, *y*−1/2, −*z*+1/2; (iii) −*x*, *y*−1, −*z*+1/2; (iv) −*x*+1/2, *y*+1/2, −*z*+1/2.
